# NLRX1 Deletion Increases Ischemia-Reperfusion Damage and Activates Glucose Metabolism in Mouse Heart

**DOI:** 10.3389/fimmu.2020.591815

**Published:** 2020-12-11

**Authors:** Hong Zhang, Yang Xiao, Rianne Nederlof, Diane Bakker, Pengbo Zhang, Stephen E. Girardin, Markus W. Hollmann, Nina C. Weber, Sander M. Houten, Michel van Weeghel, Richard G. Kibbey, Coert J. Zuurbier

**Affiliations:** ^1^ Department of Anesthesiology, the Second Affiliated Hospital of Xi’an Jiaotong University, Xi’an, China; ^2^ Laboratory of Experimental Intensive Care and Anesthesiology, Department of Anesthesiology, Amsterdam UMC, University of Amsterdam, Amsterdam Cardiovascular Sciences, Amsterdam, Netherlands; ^3^ Institut für Herz-und Kreislaufphysiologie, Heinrich-Heine Universität, Dusseldorf, Germany; ^4^ Department of Laboratory Medicine and Pathobiology, University of Toronto, Toronto, ON, Canada; ^5^ Icahn Institute for Data Science and Genomic Technology, Department of Genetics and Genomic Sciences, Icahn School of Medicine at Mount Sinai, New York, NY, United States; ^6^ Laboratory Genetic Metabolic Diseases, Amsterdam UMC, University of Amsterdam, Amsterdam Gastroenterology & Metabolism, Amsterdam, Netherlands; ^7^ Department of Cellular & Molecular Physiology, Yale University School of Medicine, New Haven, CT, United States

**Keywords:** cardiac metabolism, oxygen consumption, innate immunity, infarct size, duration of ischemia

## Abstract

**Background:**

NOD-like receptors (NLR) are intracellular sensors of the innate immune system, with the NLRP3 being a pro-inflammatory member that modulates cardiac ischemia-reperfusion injury (IRI) and metabolism. No information is available on a possible role of anti-inflammatory NLRs on IRI and metabolism in the intact heart. Here we hypothesize that the constitutively expressed, anti-inflammatory mitochondrial NLRX1, affects IRI and metabolism of the isolated mouse heart.

**Methods:**

Isolated C57Bl/6J and NLRX1 knock-out (KO) mouse hearts were perfused with a physiological mixture of the essential substrates (lactate, glucose, pyruvate, fatty acid, glutamine) and insulin. For the IRI studies, hearts were subjected to either mild (20 min) or severe (35 min) ischemia and IRI was determined at 60 min reperfusion. Inflammatory mediators (IL-6, TNFα) and survival pathways (mito-HKII, p-Akt, p-AMPK, p-STAT3) were analyzed at 5 min of reperfusion. For the metabolism studies, hearts were perfused for 35 min with either 5.5 mM ^13^C-glucose or 0.4 mM ^13^C-palmitate under normoxic conditions, followed by LC-MS analysis and integrated, stepwise, mass-isotopomeric flux analysis (MIMOSA).

**Results:**

NLRX1 KO significantly increased IRI (infarct size from 63% to 73%, end-diastolic pressure from 59 mmHg to 75 mmHg, and rate-pressure-product recovery from 15% to 6%), following severe, but not mild, ischemia. The increased IRI in NLRX1 KO hearts was associated with depressed Akt signaling at early reperfusion; other survival pathways or inflammatory parameters were not affected. Metabolically, NLRX1 KO hearts displayed increased lactate production and glucose oxidation relative to fatty acid oxidation, associated with increased pyruvate dehydrogenase flux and 10% higher cardiac oxygen consumption.

**Conclusion:**

Deletion of the mitochondrially-located NOD-like sensor NLRX1 exacerbates severe cardiac IR injury, possibly through impaired Akt signaling, and increases cardiac glucose metabolism.

## Introduction

The innate immune system is an important player in cardiac ischemia-reperfusion injury (IRI), serving as the first defense system against infection or injury through engagement of pattern recognition receptors (PRRs) such as the cell membrane-associated toll-like receptors (TLRs) and intracellular NOD-like receptors (NLRs) (1, 2). In general, activation of these receptors results in boosting a pro-inflammatory response at the place of infection or injury (1, 2). In contrast, the nucleotide-binding oligomerization domain (NOD)-like receptor X1 (NLRX1), an intracellular regulator of innate and adaptive immune response, has anti-inflammatory effects (3). It is ubiquitously expressed and mainly localized to mitochondria (3, 4). NLRX1 functions as an anti-inflammatory NLR in various disease models such as viral infection, cancer and multiple sclerosis (3–7). Considering it is a mitochondrial immune sensor, accumulating evidence implicates its potential role in mitochondrial regulated processes such as cell death and metabolism. In the present study we aimed to characterize to what extent NLRX1 affects cardiac IRI and cardiac metabolism.

NLRX1 has been found to attenuate kidney IRI (8). So far, whether NLRX1 affects cardiac IRI has only been examined in one study employing isolated non-beating cultured cardiac cells. This study demonstrated that NLRX1 reduces apoptosis and inflammatory responses following 24 h hypoxia in non-primary quiescent H9c2 cells (9). However, the effect of NLRX1 in a more physiological model of acute cardiac IRI using intact beating hearts is lacking. Reduced activation of inflammatory pathways and/or activation of cardioprotective survival proteins such as mitochondrial HKII (10–12), AMPK (13), Akt (14), and STAT3 (15) facilitate cardiac protection against IRI. We set out to determine if these mediators are involved in NLRX1 effects on IRI, by measuring them at early reperfusion, before necrosis has been finalized. Finally, because it is known that protection against cardiac IRI is critically dependent on duration of ischemia, i.e. degree of infarction (16, 17), effects of NLRX1 were examined for short and long ischemia duration. Such information is necessary for translation to the clinical condition, where ischemia severity is highly variable (18).

NLRX1 has also been found to affect metabolism. Recent studies have reported that NLRX1 may limit fatty acid oxidation (FAO) and promote glycolysis in hepatocytes from a non-alcoholic fatty liver disease (NAFLD) model (19), or reduce mitochondrial oxygen consumption in kidney epithelial cells (8). The deletion of NLRX1 protects against diet-induced metabolic syndrome and NAFLD development (19). Whether NLRX1 affects cardiac metabolism is currently unknown. However, if the reported NLRX1 effects on limitation of fatty acid oxidation and mitochondrial oxygen consumption in non-cardiac tissues also occur in the heart, this would then indicate the same metabolic signature that offers protection against cardiac IRI (20–22).

Therefore, in this study we employed the isolated Langendorff-perfused mouse heart model to address the hypothesis that NLRX1 affects cardiac IRI and cardiac metabolism.

## Materials and Methods

### Animals

NLRX1^-/-^ (NLRX1 KO) mice were generated as reported previously (23) and bred in at our institute. Age and weight -matched (11–16 weeks old, weighing 20–30 g) C57BL/6J WT mice were obtained from Charles River. Only male mice were included for the experiments. All animals were housed in standard housing conditions and had free access to food and water. All animal experiments were approved by the Animal Ethics Committee of the Academic Medical Center, Amsterdam, The Netherlands and performed in accordance with guidelines from Directive 2010/63/EU of the European Parliament on the protection of animals used for scientific purposes.

### Heart Isolation and Perfusion

Preparations were done as previously reported (24, 25). Briefly, mice were anesthetized with fentanyl (0.5 mg/kg), midazolam (9.4 mg/kg) and acepromazine (9.4 mg/kg) and heparinized (15 IU) by intraperitoneal injection. Pedal withdrawal reflex was used to ensure a surgical depth of anesthesia was obtained. Afterwards, tracheotomy and mechanical ventilation were performed. Hearts were cannulated in chest and immediately perfused. Subsequently, excised hearts were connected to a Langendorff apparatus and perfused at a constant flow, starting with an initial perfusion pressure of 80 mmHg, using Krebs-Henseleit buffer (KHB) which was filtered by 0.45 µm filter and oxygenated with 95%O_2_/5%CO_2_. The KHB contains (in mmol/L, mM) 118 NaCl, 4.7 KCl, 1.2 MgSO_4_, 1.2 KH_2_PO_4_, 25 NaHCO_3_, 0.5 EDTA, 2.50 CaCl_2_, with mixed substrates 5.5 D-glucose, 0.5 L-glutamine, 1 lactate, 0.1 pyruvate, 1%(w/v) albumin – 0.4 mM palmitic acid sodium salt, 0.05 L-carnitine and 30 mU/L insulin. Substrate concentrations were chosen to reflect *in vivo* concentrations of the non-fasted condition (26, 27). Hearts were continuously submerged in 37°C KHB. A well-stretched polyethylene balloon was inserted into the left ventricle through the pulmonary vein, filled with 20–30 µl water, to monitor left ventricular function. All hearts were initially subjected to 20 min stabilization, at the end of which perfusion pressure was set at 80 ± 5mmHg and end diastolic pressure (EDP) at 2–5 mmHg. Developed left ventricular pressure (DLVP) was calculated from peak left ventricular systolic pressure minus EDP. Rate-Pressure-Product (RPP) equaled the product of DLVP and heart rate (HR). Hearts displaying developed left ventricular pressures (DLVP) < 80 mm Hg, heart rates < 280 beats/min, irregular heartbeat or flow > 4 ml/min were excluded. From a total of 86 mice used, three were lost during anesthesia (premature dead), four were lost due to unsuccessful in-chest cannulation, four hearts did not fulfill our cardiac physiological parameters (see above), resulting in 75 successful experiments.

### Experimental Protocols

**Figure 1:**
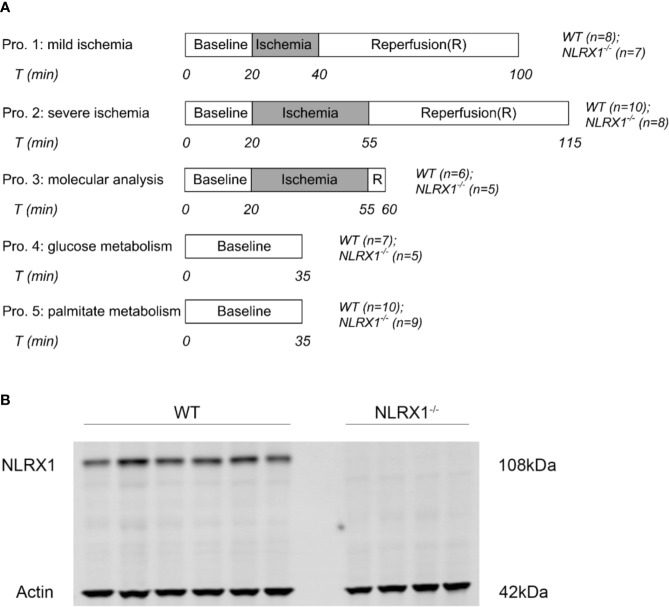
**(A)** Perfusion protocols for NLRX1 effects on IR injury, survival kinases at reperfusion and ^13^C cardiac metabolism. Hearts from WT mice and NLRX1^-/-^ mice were perfused with mixed substrates as described in methods part and subjected to 20 min baseline perfusion. For IR injury, hearts were subjected to 20 or 35 min ischemia followed by 60 min reperfusion. For measuring vital survival proteins, hearts were subjected to 35 min ischemia and 5 min reperfusion. For detecting metabolism, hearts were perfused for 35 min with either U-^13^C_6_ glucose or U-^13^C_16_ palmitate. **(B)** immunoblot results showing the presence of NLRX1 in WT hearts, and the absence of NLRX1 in NLRX1^-^/^-^ hearts.

Within each protocol (**Figure 1**), WT and NLRX1^-/-^ animals were equally distributed to time of day (morning versus afternoon) experimentation.

1. mild IRI: following 20 min baseline perfusion, hearts were subjected to 20 min global no-flow ischemia, followed by 60 min reperfusion. Perfusion and left ventricular pressures were continuously monitored (n=8 in WT group, n=7 in NLRX1^-/-^ group). After reperfusion, hearts were blotted dry, weighted, and stored at -20°C for determination of infarct size by TTC staining within 1 week.
2. severe IRI: similar as described for mild IRI, except that ischemic duration was extended to 35 min (n=10 in WT group, n=8 in NLRX1^-/-^ group).
3. molecular characterization: following 35 min no-flow ischemia and 5 min reperfusion, hearts were immediately homogenized and partly centrifuged to obtain mitochondrial, cytosolic and whole-cell fractions for determination of survival kinases (n=6 in WT group, n=5 in NLRX1^-/-^ group) and inflammatory cytokines.
4. carbohydrate metabolism: 5.5 mM non-labeled glucose in the perfusate was replaced by 5.5 mM [U-^13^C_6_] glucose (initial molar enrichment (MPE): 99%; Cambridge Isotope Laboratories, Andover, USA). After 25 min perfusion, the pulmonary artery was sampled to obtain venous oxygen tension levels [analyzed by blood gas analyzer (Siemens Rapidpoint500)]. At 35 min perfusion, hearts were immediately frozen in liquid nitrogen and stored at -80°C until further examination. Influent samples were then collected to determine arterial oxygen tension by blood gas analyzer (n=7 in WT group, n=5 in NLRX1^-/-^ group).
5. long-chain fatty acid metabolism: similar treatment as described for glucose metabolic characterization, except that 0.4 mM non-labeled palmitate was replaced by 0.4 mM [U-^13^C_16_] palmitate (initial molar enrichment (MPE): 98%; Cambridge Isotope Laboratories, Andover, USA) (n=10 in WT group, n=9 in NLRX1^-/-^ group).

### Infarct Size Measurement

The frozen hearts in IRI protocols were cut into 1mm thick slices and immersed in 1% triphenyltetrazolium chloride (TTC) solution (pH=7.4) for 20 min at 37°C on a shaker at 300 rpm. Subsequently, the slices were incubated in 4% formalin (pH = 7.4) for 2 h at room temperature and scanned afterwards. Infarct size was determined using Image J software, by an investigator not aware of treatment allocation.

### Tissue Homogenization

For the molecular characterization protocol, hearts were quickly minced in cold homogenization buffer (1.5 ml per heart) containing 0.25 M sucrose, 0.02 M HEPES, 1 mM dithiothreitol (DTT) and phosphatases and protease inhibitors on ice, and homogenized in ice-cold glass-Teflon potter at 1200 rpm/min. Then 750 μl homogenate was centrifuged at 10,000g for 10 min at 4°C to gather mitochondria in the pellet. The supernatant was the cytosolic fraction. The pellets were lysed in homogenization buffer supplemented with 0.5% triton X-100 and 1mM glucose-6-phosphate (to detach HKII from mitochondria (24)) at room temperature for 15 min. Afterwards, samples underwent sonication on ice for 4s and centrifugation (10,000 g) at 4°C for 1min. The supernatant containing the dissolved mitochondrial fraction was stored at -80 °C before determining mitochondrial hexokinase (HK) protein and enzyme activity. The remaining 750 μl homogenate was designated the whole-heart homogenate, lysed and sonicated as for the mitochondrial fraction, centrifuged for 1 min at 10,000 g and the supernatant used for determining survival pathway components in the whole heart.

Hearts immediately excised from mice (6 WT and 4 NLRX1^-/-^mice) were frozen in liquid nitrogen and stored at -80° until analysis. The whole heart was homogenized, lysed and sonicated in a similar fashion as described above for the “remaining 750 μl homogenate”. The whole-heart homogenate of these hearts was used for detection of NLRX1 by Western blot (WB) and determination of whole-heart metabolic enzyme activities.

### Enzyme Activity Assays

The maximal activities of the glycolytic enzymes LDH and HK, the mitochondrial marker enzyme CS (citrate synthase) and the fatty acid oxidation enzyme short-chain-3-hydroxyacyl-CoA dehydrogenase (SCHAD) were determined spectrophotometrically at 25°C as previously reported (28). In short, LDH activity was determined from rate of NADH oxidation at 340 nm following the addition of pyruvate. HK activity measured by the rate of NAD reduction at 340 nm in assay buffer containing glucose, glucose-6-phosphate dehydrogenase, ATP, NAD^+^ and rotenone. CS activity equaled the rate of thionitrobenzoic acid generation at 412 nm in assay medium containing acetyl CoA, oxaloacetate and di-thionitrobenzoic acid. SCHAD was determined spectrophotometrically using MES, KH2PO4, DTT, NADH, and acetoacetyl-CoA, PH 6.2. Activities were corrected for protein concentration.

### Western Blotting

NLRX1, HKII, Akt, AMPK, STAT3, and PDH proteins were determined by Western blotting. HKII was determined in cytoplasmic and mitochondrial fractions; all other proteins in whole-cell fractions. Lowry assay was used to determine protein concentration. Western blotting was conducted as described previously (29). Briefly, 10 μg protein per sample was electrophoresed on a 4–12% sodium dodecyl sulfate polyacrylamide (SDS) gel (Biorad) and transferred to polyvinylidene fluoride (PVDF) membrane. The membrane was incubated in Odyssey blocking buffer to reducing non-specific binding for 1 h. Afterwards, it was probed with the antibody for NLRX1 (1:5000; Abcam ab107611), HKII (1:5000; CST #2867), phospho-Akt (Ser473) (1:1000; CST #9271), Akt (1:1000; CST #9272), phospho-AMPKα (Thr172) (1:1000; CST #2535), AMPKα (1:1000; CST #2603), phospho-STAT3 (Thr705) (1:1000; CST #9131), STAT3 (1:1000; CST #9139), PDH (1:1000; CST #2784s), phospho-PDH E1a (Ser293) (1:2000; Sigma AP1062), and the cytosolic marker Actin (1:5000; Sigma A2066), a-tubulin (1:5000; Sigma T9026) or the mitochondrial marker COX IV (1:9000; CST #4844). Membranes were washed with PBS containing 0.1% Tween**-**20 (Sigma) and incubated with the complementary secondary fluorescence antibody (IRdye, Licor, Lincoln, USA, 1:5000; #926-68071/926-32211 or 926-32210) for 1 h at room temperature before they were washed again and scanned with an Odyssey scanner(Li-cor). Within one blot, the intensity of all bands was normalized to band with highest intensity.

### Cytokine Determination in Hearts

IL-6 and TNFα were determined in the supernatant of the 10,000 g 10 min centrifuged whole heart homogenized tissue, using ELISA kits (#DY406 and #DY410, R&D Systems) following manufacturer’s instructions. Final quantification was normalized by whole protein concentration in the samples, as determined by Lowry assay.

### LC-MS

Metabolomics was performed as previously described, with minor adjustments (30). Samples were freeze dried, crunched and approximately 2 mg weighted in a 2 ml tube. A 75 µl mixture of internal standard adenosine-^15^N_5_-monophosphate (100 µM) was added to each sample. Subsequently, 425 µl water, 500 µl methanol and 1 ml chloroform were added to the same 2 ml tube before thorough mixing and centrifugation for 10 min at 14.000 rpm. The top layer, containing the polar phase, was transferred to a new 1.5 ml tube and dried using a vacuum concentrator at 60°C. Dried samples were reconstituted in 100 µl methanol/water (6/4; v/v). Metabolites were analyzed using a Waters Acquity ultra-high-performance liquid chromatography system coupled to a Bruker Impact II™ Ultra-High Resolution Qq-Time-Of-Flight mass spectrometer. Samples were kept at 12°C during analysis and 5 µl of each sample was injected. Chromatographic separation was achieved using a Merck Millipore SeQuant ZIC-cHILIC column (PEEK 100 x 2.1 mm, 3 µm particle size). Column temperature was held at 30°C. Mobile phase consisted of (A) 1:9 acetonitrile:water and (B) 9:1 acetonitrile:water, both containing 5 mM ammonium acetate. Using a flow rate of 0.25 ml/min, the LC gradient consisted of: 100% B for 0-2 min, ramp to 0% B at 28 min, 0% B for 28–30 min, ramp to 100% B at 31 min, 100% B for 31–35 min. Mass spectrometry (MS) data were acquired using negative and positive ionization in full scan mode over the range of m/z 50–1200. Data were analyzed using Bruker TASQ software version 2.1.22.3. Isotope ratios and correction for contribution of naturally occurring isotopes were calculated using IsoCorrectoR (31). Metabolite identification has been based on a combination of accurate mass, (relative) retention times and fragmentation spectra, compared to the analysis of a library of standards.

### MIMOSA Analysis

For further analysis and interpretation of labeled substrates we applied mass isotopomer multi-ordinate spectral analysis (MIMOSA), as a stepwise flux analysis program to estimate relative glycolytic and mitochondrial rates (32). To this end, MIMOSA uses the transfer of the ^13^C label going from the precursor to the product of each metabolic conversion, to obtain and compare the estimates of rates of each such conversion between wild-type and NLRX1^-/-^ hearts. The positional assignments of enrichments were calculated from the isotopologue data (without carbon-carbon breakage) from simplifications in isotope patterns from the primarily oxidative metabolism of the perfused hearts (see **Supplementary Table 1**). When glucose was labeled, the high glycolytic intermediate precursor enrichment (>95%) all the way through PEP with <1% M+1 or M+2, no significant M+1 AcCoA, and the absence of significant oxidative pentose metabolism (no enrichment in ribulose-5-phosphate), indicates limited dilution or scrambling of glycolytic label. Additionally, this also rules out significant PEP cycling through PEPCK (data not shown). In the presence of an unresolvable isobar with M+0 succinate, the similarity of the M+3/(M+2 + M+4) ratios in succinate, fumarate, and malate (not shown) indicates that there is no interfering pyruvate carboxylase (PC) flux under the conditions used. With glucose as label, the absence of pyruvate cycling through malic enzyme was ruled out by the absence of significant M+1 or M+2 pyruvate or lactate. Anaplerotic and cataplerotic fluxes were also largely ruled out by lack of any heavy isotopes of pyruvate, acetate or PEP when labeled palmitate was used. Consequently, there is equal probability of each of the four M+3 isotopomers of oxaloacetate giving rise to citrate.

Having ruled out any M+1 acetyl CoA, all heavy citrate isotopomers are a consequence of the ligation of either M+0 or M+2 acetyl carbons with the isotopomers of oxaloacetate. Since, acetyl CoA does not equilibrate across both the mitochondrial and cytosolic compartments, five independent mass isotopomer distribution analyses (MIDA) were performed to identify the enrichment of the mitochondrial acetyl CoA (**Supplementary Table 1**; those involving M+3 citrate were not included because of low signal to noise). The 5 independent calculations gave remarkably similar answers for the enrichment of mitochondrial acetyl CoA (not shown). As such, the labeled carbon derived from the PDH reaction could be followed around the TCA cycle into citrate, alpha-ketoglutarate, and malate from the straightforward algebraic deconvolvement of the isotopologues. For instance, the sum of heavy citrate isotopomers M+2 or greater was adjusted by removing the contributions of ligations of M+2, M+3, and M+4 oxaloacetate/malate with M+0 acetyl CoA.

When metabolic pathways intersect, steady state ϕ calculations take advantage of the differences in enrichment between a metabolite precursor and its enzymatic product to determine the relative contribution of fluxes to the pathway (30). Cohorts of positional enrichments were compared between precursor (denominator) and product (numerator) using the relationships described in **Supplementary Table 2**.

### Statistics

**Figure 2:**
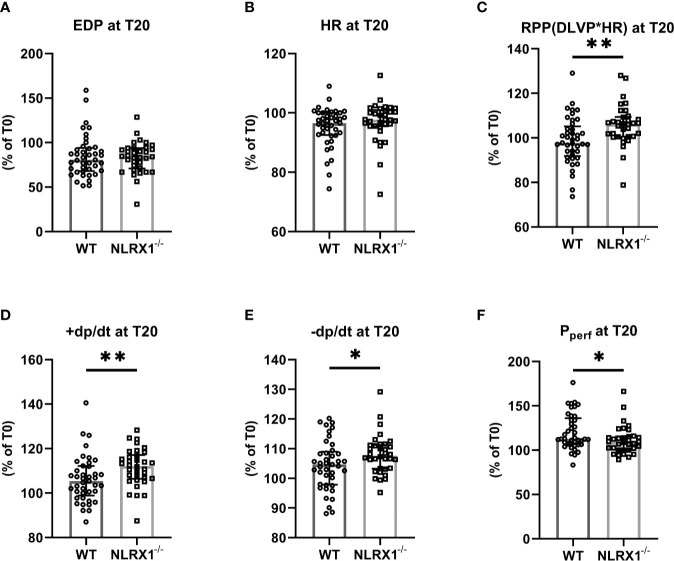
The impact of NLRX1 deletion on maintenance of mechanical function during 20 min normoxic perfusion in the isolated hearts of protocols 1 to 5. For all parameters, the change in parameter at T=20 min relative to the value at T= 0 min is depicted. **(A)** end diastolic pressure (EDP), **(B)** heart rate (HR), **(C)** Rate Pressure Product (RPP), **(D)** maximum contraction rate of left ventricle (+dp/dt), **(E)** maximum relaxation rate of left ventricle (-dp/dt), and **(F)** Perfusion pressure (P_perf_). DLVP, developed left ventricular pressure. All values represent median ± IQ (n=41 in WT group; n=34 in NLRX1^-/-^ group). *P < 0.05, **P < 0.01 by Mann-Whitney *U* test.

Data are expressed as mean ± SD, except data in **Figure 2** which are median ± IQ. Tests of normality were analyzed by Shapiro–Wilk test. Statistical comparisons were performed with unpaired Student’s *t* test when data were normally distributed, otherwise Mann–Whitney *U* test were applied. Two-way ANOVA followed by LSD test was performed when analyzing ^13^C transit and flux contribution. Possible outliers were identified using Grubbs’s test for outliers. Power calculations for the IRI studies (80% power at 5% significance to detect a 30% change) indicated that seven experiments were adequate for each parameter to reach significance. Statistics were conducted using IBM SPSS statistics version 24 (International Business Machines Corp., Armond, NY, USA). Figures were made in GraphPad Prism 8.0 (GraphPad Software, Inc., La Jolla, CA, USA). A value of *P* < 0.05 indicates statistical difference.

## Results

### NLRX1 Deletion Enhanced Mechanical Function During Normoxic Perfusion

We first confirmed that hearts from WT animals showed presence of NLRX1, whereas no NLRX1 is detected in hearts of the NLRX1^-/-^mice (**Figure 1B**). No difference in baseline cardiac performance at T0 was present between NLRX1^-/-^ and WT in our model (**Table 1**). Subsequently, hearts were left alone and perfused at constant flow for the next 20 min. NLRX1 deletion, compared to control, was without effect on EDP and heart rate development after 20 min baseline perfusion (T20) relative to T0 (**Figures 2A, B** **)**. However, NLRX1 deletion improved cardiac function, as seen by small but significant increase in RPP (% relative to T0, WT 98 ± 11, NLRX1^-/-^ 106 ± 9, *P*<0.01, **Figure 2C**), +dp/dt (% relative to T0, WT 106 ± 10, NLRX1^-/-^ 112 ± 9, *P*<0.01, **Figure 2D**) and –dp/dt at (% relative to T0, WT 105 ± 8, NLRX1^-/-^ 108 ± 7, *P*<0.05, **Figure 2E**). Additionally, NLRX1 ablation reduced the degree of vasoconstriction when compared to WT hearts (**Figure 2F**). These results indicate that the presence of NLRX1 impairs cardiac mechanical performance.

**Table 1:** Baseline functional values at T0 in WT and NLRX1^-/-^ isolated mouse hearts from protocols 1 to 5.

	WT	NLRX1^-/-^
P_perf_ (mmHg)	81 ± 9	82 ± 12
EDP (mmHg)	3.1 ± 1.3	3.6 ± 1.6
DLVP (mmHg)	129 ± 17	130 ± 13
HR (beats/min)	348 ± 37	342 ± 36
RPP (DLVP*HR)	44875 ± 7177	44421 ± 6875
+dp/dt (mmHg/s)	5070 ± 792	4833 ± 652
-dp/dt (mmHg/s)	4281 ± 491	4215 ± 433
Flow (ml/min)	2.1 ± 0.6	2.0 ± 0.4

No difference was found in baseline characteristics between WT and NLRX1^-/-^ groups. Values represent means ± SD (n=41 in WT group and n=34 in NLRX1^-/-^ group). P_perf_, perfusion pressure; EDP, end diastolic pressure; DLVP, developed left ventricular pressure; HR, heart rate; RPP, rate pressure product; +dp/dt, maximum contraction rate of left ventricle; and -dp/dt, maximum relaxation rate of left ventricle.

### NLRX1 Deletion Increased Severe IR Injury, Without Affecting Mild IR Injury

**Figure 3:**
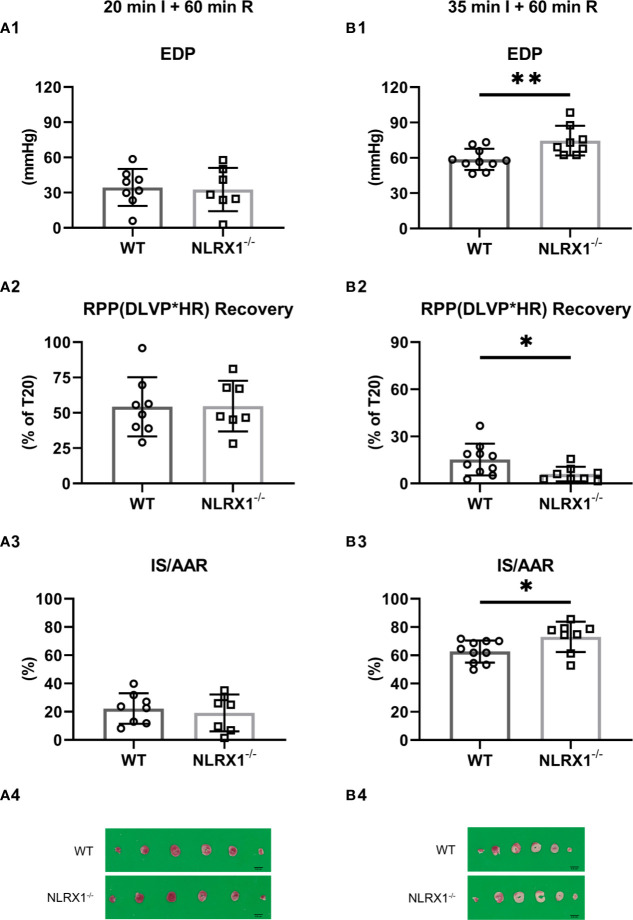
Effects of NLRX1 deletion on mild **(A)** and severe **(B)** IR injury in isolated hearts of protocols 1 and 2. All values determined at 60 min reperfusion. (A1/B1) End diastolic pressure (EDP); (A2/B2) Rate Pressure Product (RPP) recovery; and (A3/B3) Infarct size (IS). DLVP, developed left ventricular pressure; HR, heart rate; AAR, area at risk. Values represent mean ± SD. (n=8/7 per group for **(A)**; n=10/8 per group for **(B)**. *P < 0.05, **P < 0.01 by *t* test.

We evaluated whether NLRX1 influenced IR injury in isolated hearts (**Figure 3**). First, we examined a mild ischemic insult (20 min; **Figure 3A**). At the end of 60 min reperfusion, EDP was 34 ± 16 mmHg, Rate-Pressure-product (RPP) recovered to 46 ± 11%, and infarct size was 22 ± 11% for WT (**Figures 3A1–A3**). Similar IRI outcome parameters were observed for the NLRX1^-/-^ hearts. Extending the ischemic period to 35 min largely increased damage to the heart. At the end of reperfusion in the WT hearts, EDP was now 59 ± 9 mmHg, RPP recovered to 15 ± 10% only, with an infarct size of 63 ± 8% (**Figures 3B1–B3**). NLRX1 deletion, modestly but significantly, worsened IR injury, as reflected by an increased EDP (75 ± 13 mmHg), decreased %RPP (6 ± 5%) and enlargement of infarct size (73 ± 11%, *P*<0.05). Thus, the presence of NLRX1 protects against severe but not mild IRI.

### NLRX1 Deletion Reduced Activation of Akt Signaling at Early Reperfusion in Severe IR Injury

**Figure 4:**
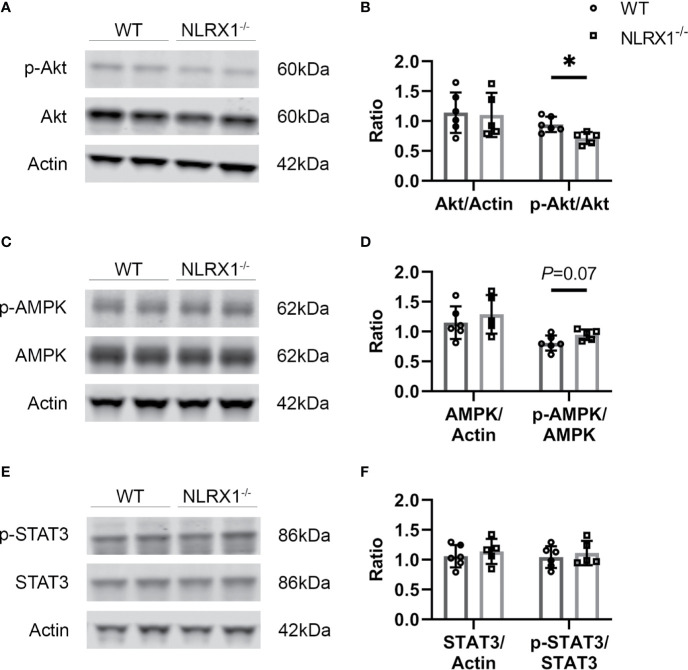
Survival signaling pathways and inflammatory factors at early stage of reperfusion in severe IRI model of WT and NLRX1^-/-^ isolated mouse hearts of protocol 3. **(A, B)** Representative immunoblots and analysis of total Akt and phospho-Akt; **(C, D)** Representative immunoblots and analysis of total AMPK and phospho-AMPK; **(E, F)** Representative immunoblots and analysis of total STAT3 and phospho-STAT3. Values represent mean ± SD. (n=6/5 per group). *P < 0.05 by *t* test.

We investigated whether NLRX1 effects on severe IR injury could be associated with changes in cardiac survival signaling pathways at early reperfusion (**Figure 4**). NLRX1 deletion significantly reduced Akt phosphorylation at Ser473 site compared with WT group (WT 1.09 ± 0.15, NLRX1^-/-^ 0.83 ± 0.12, *P*=0.01, **Figures 4A, B**). AMPK phosphorylation at Thr172 site was not increased in the NLRX1^-/-^ group compared with WT group (WT 0.81 ± 0.13, NLRX1^-/-^ 0.95 ± 0.09, *P*=0.07, **Figures 4C, D**). There was no difference in STAT3 phosphorylation at Thr705 site in the NLRX1^-/-^ group compared with WT group (WT 1.04 ± 0.18, NLRX1^-/-^ 1.11 ± 0.20, *P*=0.57, **Figures 4E, F**). No differences in p-Akt or p-STAT3 were detected in hearts immediately excised from the animals (**Supplementary Figure 1**). Next, we examined whether NLRX1 deletion affected mtHK at early reperfusion (**Supplementary Figure 2**). There was no significant difference in cytosolic HK activity (**Figure 1A** **Suppl**) or mtHK activity (**Figure 1B** **Suppl**). In addition, no differences were observed in cytosolic HK II (**Figures 1C** **, E Suppl**) or mtHKII amount (**Figures 1D** **, F Suppl**) between NLRX1^-/-^ group and WT group. Finally, analysis of cardiac inflammatory cytokines at early reperfusion demonstrated similar levels of IL-6 and TNFα between the two genotypes (**Figures 1G** **, H Suppl**).

### NLRX1 Deletion Increased Lactate Generation and Glucose Oxidation Through Increased PDH Flux

**Figure 5:**
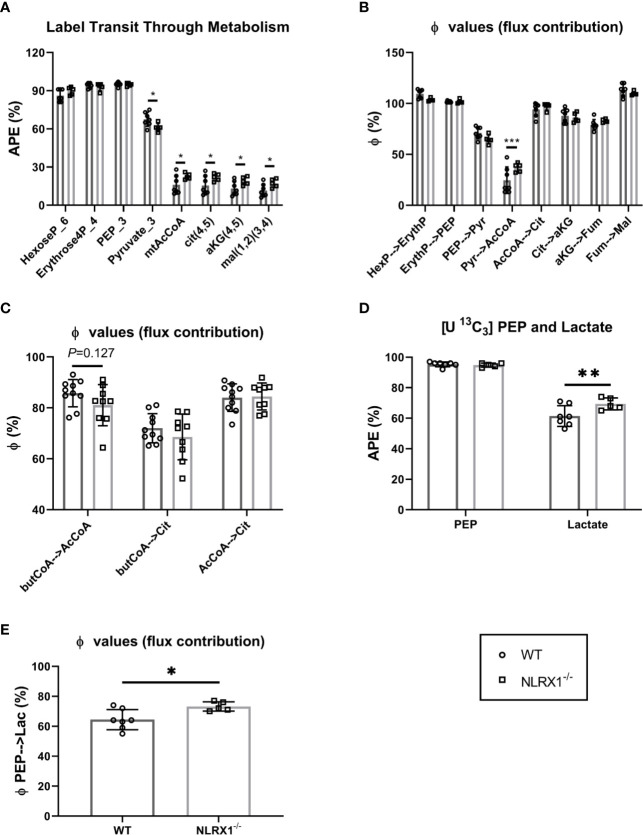
Metabolism of isolated beating WT and NLRX1^-/-^ hearts characterized following 35 min perfusion with U-^13^C_6_ labeled 5.5 mM glucose or U-^13^C_16_ 0.4 mM palmitate. **(A)** Metabolite sequential enrichment in glycolysis (hexose-6-phosphate, PEP, pyruvate), pentose phosphate pathway (erythrose-4-phosphate), pyruvate dehydrogenase (PDH) complex reaction (mtAcCoA) and TCA cycle (citrate, α-ketoglutarate and malate) from ^13^C glucose. The mitochondrial enrichments reflect the calculated contributions of carbons coming from acetyl CoA in the first turn; **(B)** Flux contribution (ϕ;), depicted as the ratio of precursor to product enrichment, determined by MIMOSA analysis along central carbon metabolism with each “parent” metabolite giving rise to its respective direct or clustered isotopomer(s); **(C)** Flux contribution of the ^13^C palmitate-derived labeled butyrylCoA into acetylCoA or citrate, and acetylCoA into citrate. **(D)** ^13^C labeling in PEP and lactate; **(E)** Flux contribution of the glycolytic intermediate PEP to lactate as proxy for lactate generation; APE(%), atomic percent enrichment (=(labeled isotopomer/total metabolite) * 100); PEP = phosphoenolpyruvate, Values represent mean ± SD (n= 5-10 per group). *P < 0.05, **P < 0.01, ***P < 0.001, by two-way ANOVA followed by LSD test or by *t* test.

The sequential enrichments of metabolites in the pathway from glucose through glycolysis through the PDH reaction and into and around the TCA cycle are shown (**Figure 5A**). There are no differences in steady state enrichment between NLRX1^-/-^ and control (WT) through glycolysis up to PEP. Despite identical M+3 PEP enrichments, the pyruvate enrichment was lower in knockouts indicating a dilutive contribution from an unlabeled source (e.g., transamination of alanine, pyruvate or lactate uptake, malic enzyme exchange). Even with lower pyruvate enrichment compared to control, acetyl CoA and all subsequent mitochondrial metabolites were more enriched relative to controls suggesting a greater contribution of pyruvate oxidation relative to beta-oxidation in knockouts.

Along a metabolic pathway the enrichment of a product metabolite at metabolic steady state is dependent on the enrichment of its precursor. If the positional (isotopomeric) enrichment of precursor and product are the same, then the precursor is the only pathway contributing to the synthesis of the product. Dilutions in the product enrichment relative to the precursor indicate contributions from other unlabeled pathways. For instance, in the heart pyruvate, beta-oxidation, acetate, and ketones all can contribute to the synthesis of mitochondrial acetyl CoA and so if pyruvate is the only source of label, the dilution of acetyl CoA relative to pyruvate indicates the contribution of carbohydrate oxidation to the TCA cycle. In the controls, the enrichment of mitochondrial acetyl CoA relative to pyruvate (ϕ;pyr→AcCoA) was ~20% indicating that in these perfused hearts, carbohydrates only contributed one fifth of the oxidized carbon through the pyruvate dehydrogenase (PDH) complex. Remarkably, except for the enrichment in acetyl CoA coming from pyruvate, the precursor-product relationships (ϕ;) were identical throughout glycolysis and the TCA cycle (**Figure 5B**). This indicates the observed differences in enrichment in central carbon metabolism between control and NRLX1^-/-^ can all be attributed to an increase in pyruvate oxidation. Because of the potential contributions of acetate and ketones to non-carbohydrate oxidation at this step, the ^13^C palmitate perfusions provide a near mirror image to the labeled glucose experiments (**Figure 5C**). Even so, a trend consistent with increased pyruvate oxidation was shown by the diminished contribution of butyryl-CoA to both acetyl CoA and citrate for NLRX1^-/-^ hearts. Thus, deletion of NLRX1 within the heart resulted in increased mitochondrial PDH-derived acetyl CoA oxidation relative to fatty acid β-oxidation.

In addition to mitochondrial oxidation, glucose can contribute to ATP production by fermentative glycolysis to generate lactate. Since the perfusate also contained unlabeled lactate at a constant concentration, it can exchange across the cardiac monocarboxylate transporter (MCT) at a rate that is a function of glycolytic lactate exit. The premise we used for analysis of this latter part of glycolysis is that lactate M+3 enrichment is a function of the rate of PEP enrichment flowing through pyruvate kinase into lactate dehydrogenase, and that in steady state an increased lactate enrichment reflects increased LDH flux in the direction of lactate generation. Thus, a higher enrichment of heavy lactate (relative to its precursor phosphoenolpyruvate, PEP) is qualitatively indicative of a greater rate of fermentative glycolysis (i.e., there will be less dilution from external unlabeled lactate). With similar PEP enrichments, lactate enrichments were higher in NRLX1^-/-^ heart and the ϕ;PEP→Lactate was also higher (**Figures 5D, E**). Taken together, these data suggest an overall increase in the glycolytic rate in NRLX1^-/-^ hearts supporting both oxidative and fermentative ATP production.

### NLRX1 Deletion Increased Oxygen Consumption Without Alterations in Specific Enzyme Activities

**Figure 6:**
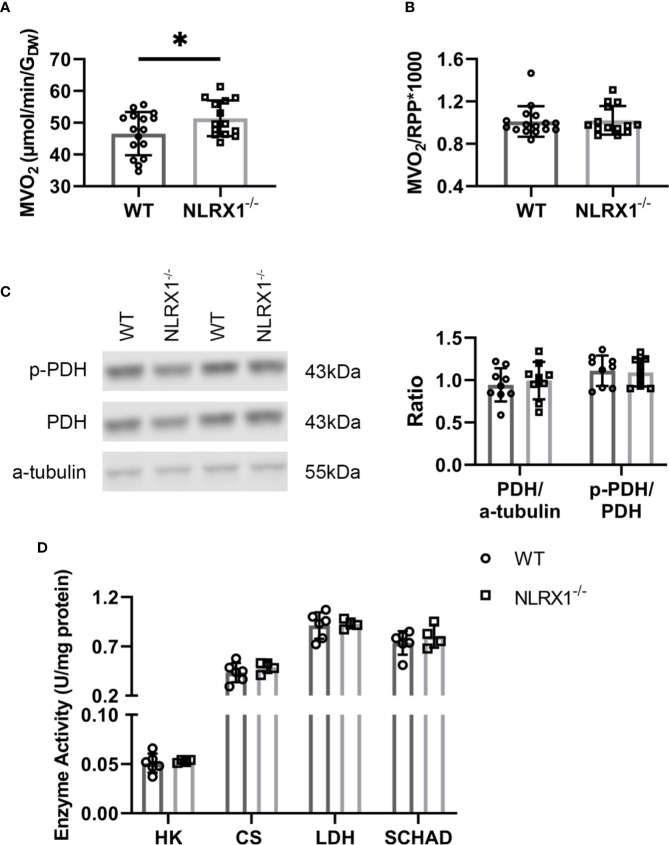
Cardiac oxygen consumption and key metabolic enzymes in WT and NLRX1^-/-^ mouse hearts. **(A)** Myocardial oxygen consumption rate (MVO_2_) and **(B)** MVO_2_/RPP at 25 min baseline perfusion (n=14-16 per group); **(C)** Total pyruvate dehydrogenase (PDH) relative toα-tubulin, and phospho-PDH (p-PDH) in hearts of both genotypes (n=9 per group); **(D)** Cardiac enzyme activities for hexokinase (HK), citrate synthase (CS), lactate dehydrogenase (LDH) and short chain 3-hydroxyacyl-CoA dehydrogenase (SCHAD) (n=4–6 per group). RPP, rate pressure product. Values represent mean ± SD. *P < 0.05 by *t* test.

To further characterize whether these changes in glucose metabolism precipitates in changes of overall energy metabolism, cardiac oxygen consumption following 25 min of perfusion of the hearts was determined. Hearts without NLRX1 displayed a 10% higher cardiac MVO_2_ as compared to WT hearts (**Figure 6A**). However, oxygen consumption relative to RPP was not altered (**Figure 6B**), indicating that the increase in oxygen consumption is matched to increased isometric work generated by the NRLX1^-/-^ hearts (see also **Figure 2C**). Therefore, NLRX1 deletion did not affect cardiac economy.

To examine whether the increased carbohydrate oxidation in the NRLX1^-/-^ hearts was associated with increased PDH activation, we semi-quantitatively determined phosphorylated PDH by western blot techniques in hearts of the ^13^C palmitate series (**Figure 6C**). Total PDH and phosphorylated PDH was without significant changes between genotypes. Finally, NLRX1 deletion was without any effect on other marker enzyme activities of lactate metabolism (LDH), glycolysis (HK), mitochondrial metabolism (CS) and fatty acid oxidation (SCHAD) (**Figure 6D**).

## Discussion

In this study we have made several novel observations concerning the role of the mitochondrial innate immune receptor NLRX1 in cardiac tissue, i.e., NLRX1 deletion 1) worsens severe, but not mild, acute IRI, 2) impairs Akt signaling at early reperfusion without changes in other survival protein or inflammatory mediators, 3) increases glucose oxidation relative to fatty acid oxidation with PDH as key regulatory step affected by NLRX1, 5) elevates lactate generation, and 4) increases oxygen consumption and cardiac mechanical function.

NLRX1 is known to function as a negative NLR, attenuating the inflammatory response to bacterial or viral infection (33). It has also been shown to reduce epithelial cell apoptosis and mitochondrial injury during *in vivo* renal ischemia-reperfusion in mice (8), and reduce cell damage following 24 h hypoxia in H9c2 cells (9). H9c2 cells are undifferentiated, non-contracting, myoblast cells, deviating largely from the beating differentiated adult heart, raising the question to what extent the observed results translate to the intact heart. The current study shows that NLRX1 protects the intact heart against acute IRI. This protection by NLRX1 against acute cardiac IRI seems unrelated to the inflammatory response of the heart, because at early reperfusion no differences in cardiac IL-6 and TNFα between WT and NLRX1 deletion were observed. Ambiguity prevails concerning NLRX1 effects on these cytokines: some studies report decreased IL-6 or TNFα with NLRX1 expression (8, 34), and other studies show no effect (19). For cardiac cells it was suggested that NLRX1 effects on cytokines were mediated through inhibition of NLRP3 expression (9). When NLRP3 is indeed the mediator of NLRX1 effects on cytokines, the very low expression of NLRP3 in healthy hearts (1, 35) can then explain why NLRX1 was without effect on cytokines in the present study. However, during longer reperfusion (hours to days) NLRX1-attenuating effects on inflammation may start to contribute to protection against IRI and remodeling, since cardiac NLRP3 expression increases not till several hours after an acute IR insult (1, 2).

Of the various intracellular survival signaling pathways known to play a causal role in acute cardiac IRI (36) and examined in the present study, NLRX1-ablation induced increased IRI was only associated with decreased phosphorylation of the survival kinase Akt. These differences in p-Akt were absent in non-IR hearts, indicating differences are because of NLRX1 effects on cardiac IRI. However, we cannot further differentiate whether these changes in p-Akt with NLRX1 ablation are specific for severe ischemia only, and not for mild ischemia, because Akt was not determined following mild ischemia. It remains unknown, however, whether NLRX1 directly activated Akt, or whether Akt activation in the presence of NLRX1 is more indirectly, and a consequence of other cellular cardioprotective processes mediated by NLRX1 during IR. An indirect effect seems more likely, given that cancer studies reported decreased Akt activation with increased forced NLRX1 expression (37, 38). It is therefore more likely that the decreased Akt phosphorylation with NLRX1 ablation is a consequence of other NLRX1-ablation induced detrimental mechanisms. One such detrimental mechanism in terms of increasing cardiac IRI may be the increase in energy metabolism in the NLRX1 KO hearts.

At similar energy production, a shift towards glucose metabolism, away from fatty acid metabolism, is generally considered as a cardioprotective strategy against cardiac IRI (22). As such, the increased glucose metabolism in the NLRX1 KO hearts is not commensurate with increased IRI in these hearts. However, we demonstrate that this increased glucose metabolism in the KO hearts is associated with a parallel increase in oxygen consumption, i.e. energy production is actually increased. It is this increase in energy turnover that could be causing the increase in cardiac IRI. Additionally, although fatty acid metabolism was only non-significantly decreased in NLRX1 ablated hearts, it is possible that there is increased accumulation of FA intermediates with diminishing fatty acid metabolism in the KO hearts. Interestingly, such increased accumulation will then also negatively affect Akt signaling (39–41). However, this remains rather speculative and warrants further research, as acylcarnitine levels in the NLRX1 KO heart have not be determined as of yet.

Previous studies reported that NLRX1 deletion resulted in either increased oxygen consumption and fatty acid oxidation in cultures of immortalized non-functional epithelial (8) and hepatocyte cells (19), or increased glycolysis and lactate metabolism, together with elevated LDH activities but without changes in oxygen consumption, in CD4^+^ T cells (42). Our findings show that the metabolic effects of NLRX1 are likely organ/tissue-specific, because for the heart loss of NLRX1 resulted in increased oxygen consumption, lactate generation and glucose oxidation.

However, the overall effect of loss of NLRX1 is always an activation of metabolism independent of cell type or organ. The increased oxygen consumption upon NLRX1 loss appears to be independent of changes in mitochondrial content, as citrate synthase activity was unaffected. Cardiac oxygen consumption may also be increased through loss of mitochondrially-bound HKII (24). However, mtHKII was also unaffected by NLRX1. Although our current data indicates that NLRX1 affects the intrinsic working of mitochondria, possible through PDH inhibition, further research is warranted to elucidate in more molecular details how NLRX1 regulates mitochondrial function. A general function of NLRX1 is providing an endogenous brake on metabolism. This metabolic brake function of NLRX1 may in itself partly explain its protective action against IRI, given that metabolic slowing protects against reperfusion injury (20, 22). One mechanism through which increased mitochondrial metabolic activity in the NLRX1 KO hearts may contribute to increased reperfusion injury, is that such increased mitochondrial activity may result in increased ROS production upon reperfusion. Indeed, ablation of NLRX1 was shown to increase ROS production following kidney IRI (8). Subsequent cardiac studies are needed to examine whether ROS is also increased in NLRX1 KO hearts subjected to IRI.

The observed increase in cardiac oxygen consumption with loss of NLRX1 was matched by an increased performance of the heart. In other words, NRX1 does not change cardiac efficiency. The increased lactate generation could not be explained by increased lactate dehydrogenase enzyme activity in the NLRX1 depleted heart, and further research will be needed to explore underlying mechanisms.

In conclusion, the results suggest that ablation of the mitochondrial NOD-like receptor NLRX1 exerts a detrimental effect on acute cardiac infarction induced by a prolonged ischemia-reperfusion episode and activates cardiac glucose metabolism and overall energy metabolism. The increase in glucose metabolism seems to be located at the PDH metabolic step. Potential IRI mechanisms contributing to increased IRI due to NLRX1 ablation, relate to elevated energy metabolism and diminished Akt. As NLRX1 seems to be downregulated in the heart from acute myocardial infarction (AMI) (9), the activation of NLRX1 by novel compounds (34) may offer new therapeutic option to protect patients from myocardial infarction.

## Data Availability Statement

The raw data supporting the conclusions of this article will be made available by the authors, without undue reservation.

## Ethics Statement

The animal study was reviewed and approved by Animal Ethics Committee of the Academic Medical Center, Amsterdam, Netherlands, and performed in accordance with the guidelines from Directive 2010/63/EU of the European Parliament on the protection of animals used for scientific purposes.

## Author Contributions

HZ and CZ designed the study. HZ, YX, and DB performed the experiments. HZ, YX, RN, SG, SH, MW, RK, and CJZ provided materials, performed measurements, and analyzed the data. HZ and CZ wrote the manuscript. RN, PZ, MH, NW, SH, and MW revised critically the manuscript. All authors contributed to the article and approved the submitted version.

## Funding

This work was supported by the National Institutes of Health/National Institute of Diabetes and Digestive and Kidney Diseases R01 DK 108283 to RK and the Chinese Scholarship Council (201706280114 and 201806270257).

## Conflict of Interest

The authors declare that the research was conducted in the absence of any commercial or financial relationships that could be construed as a potential conflict of interest.
