# Supplementary material for: NLRX1 Deletion Increases Ischemia-Reperfusion Damage and Activates Glucose Metabolism in Mouse Heart
Source: Front Immunol. 2020 Dec 11;11:591815. doi: 10.3389/fimmu.2020.591815 (PMC7759503; doi:10.3389/fimmu.2020.591815)
Supplement: Supplementary file 1 [file Presentation_1.pptx]

## Slide 1
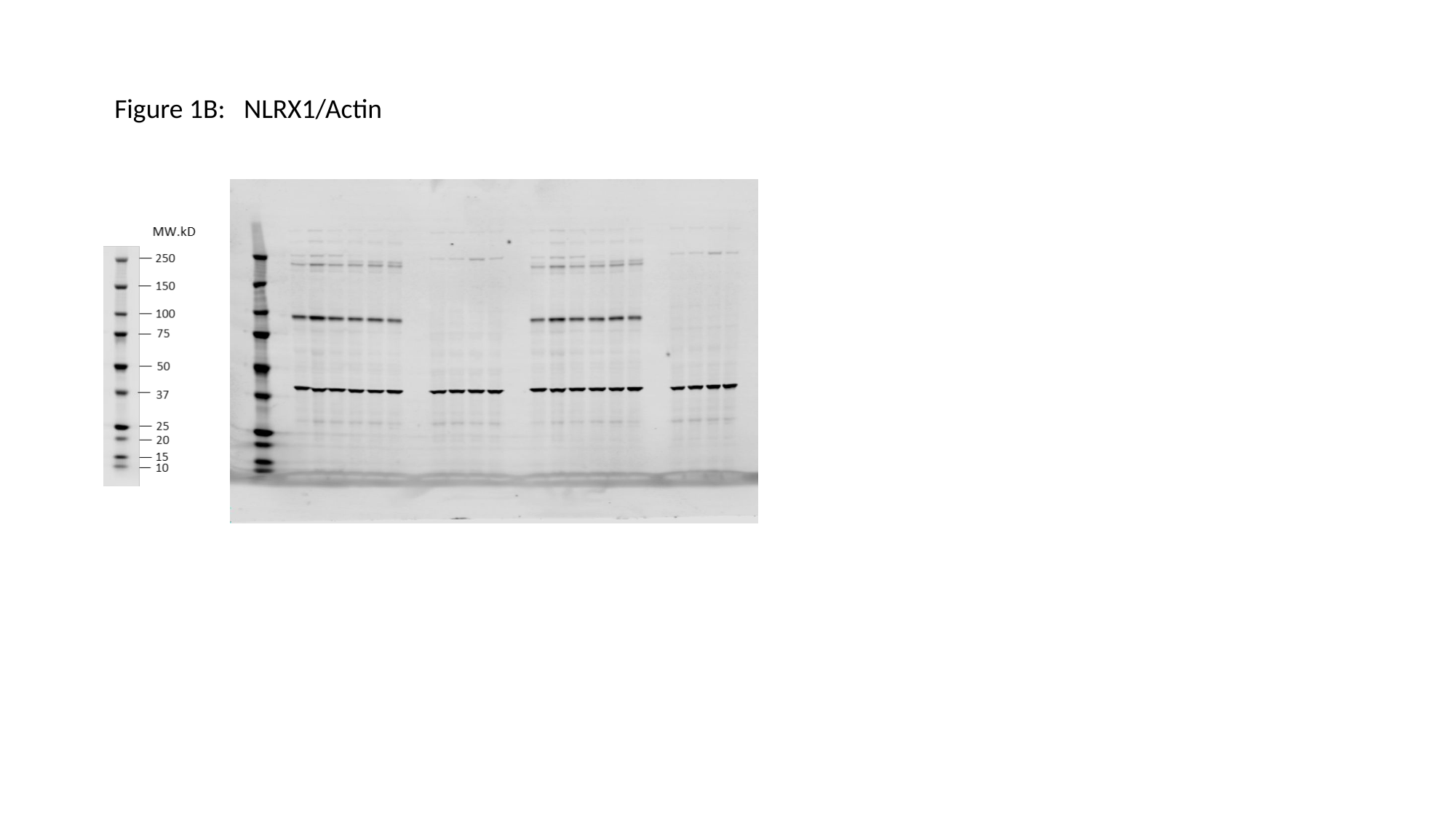

Figure 1B: NLRX1/Actin

## Slide 2
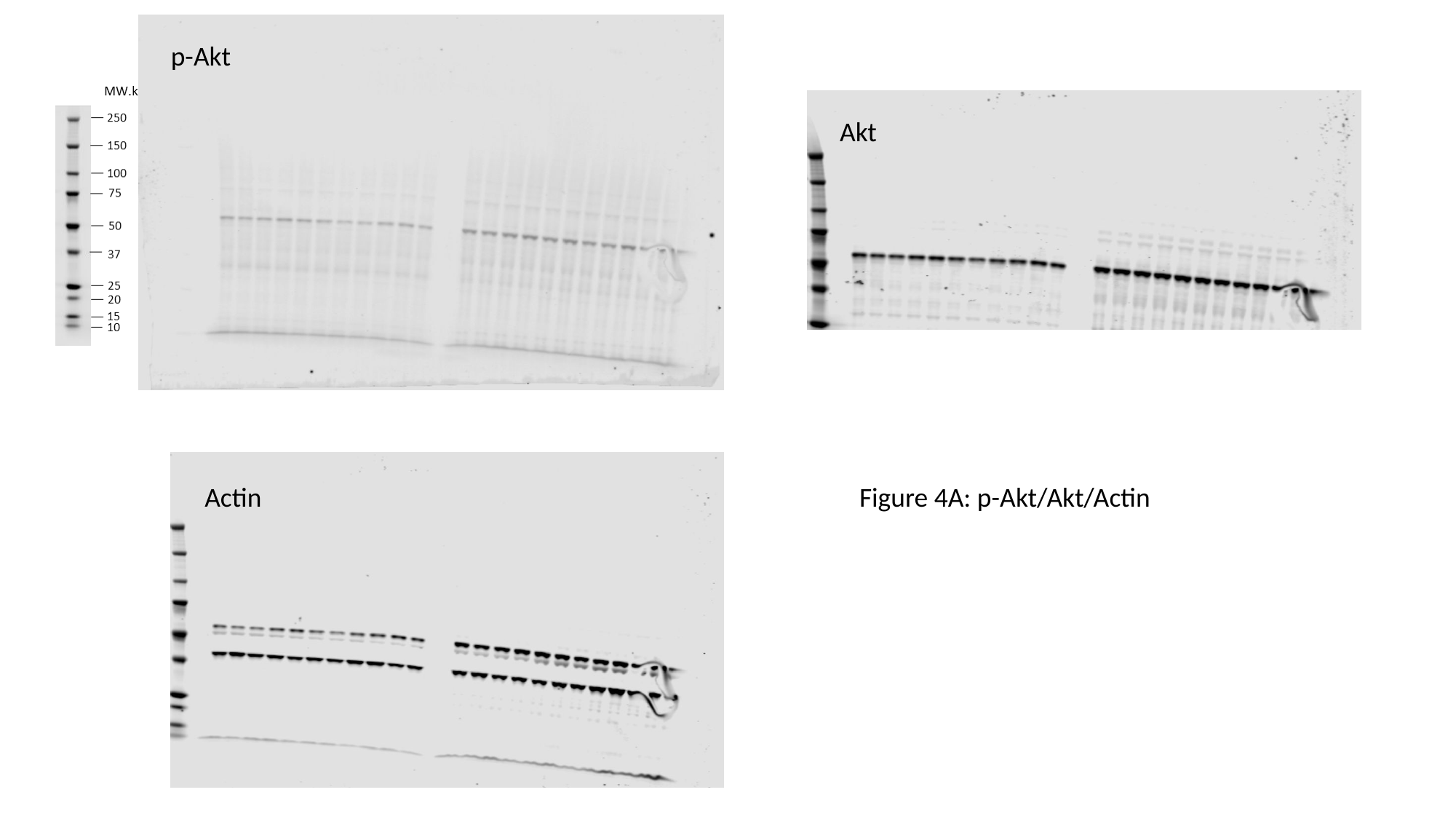

p-Akt
Akt
Actin
Figure 4A: p-Akt/Akt/Actin

## Slide 3
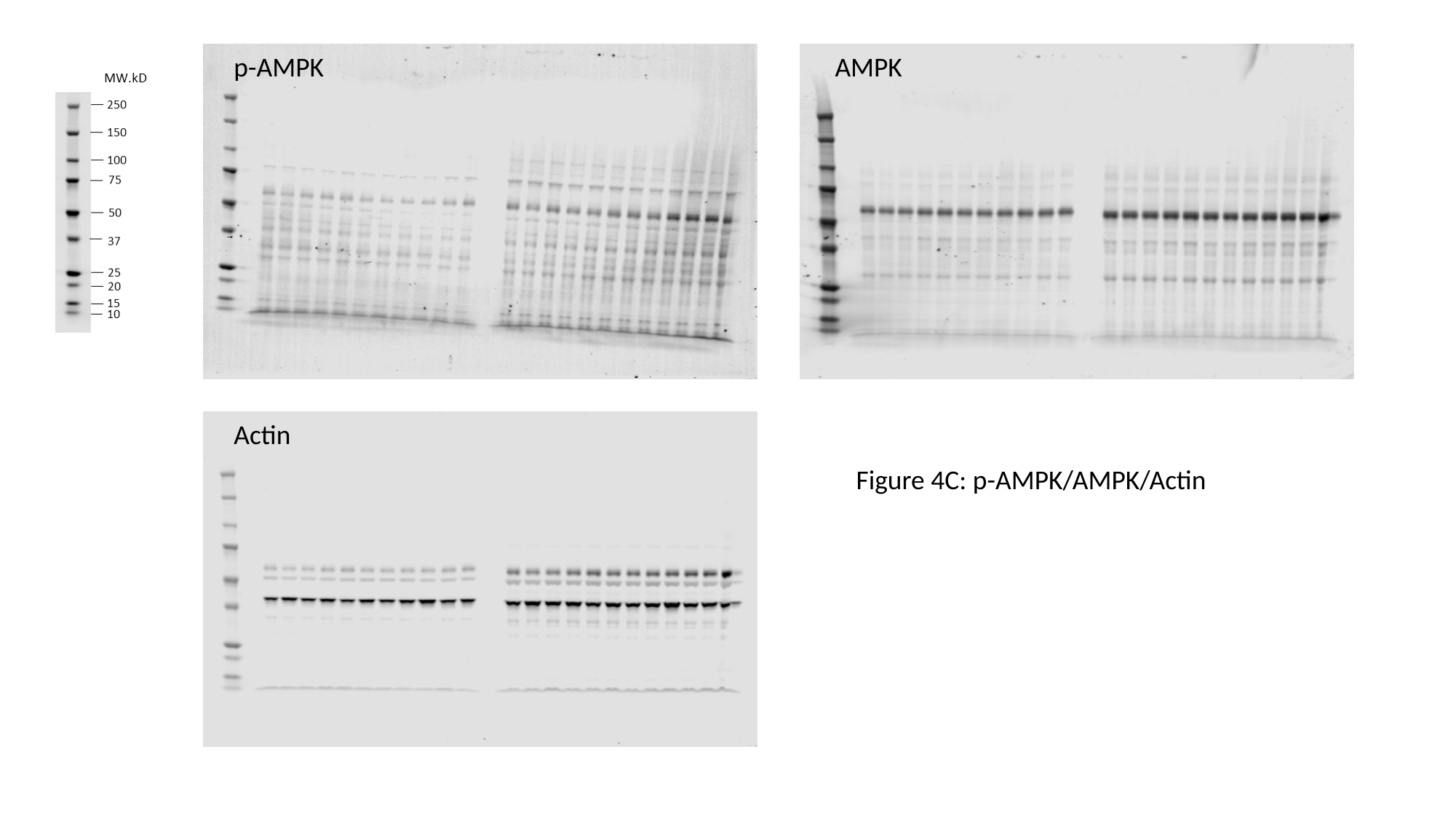

p-AMPK
AMPK
Actin
Figure 4C: p-AMPK/AMPK/Actin

## Slide 4
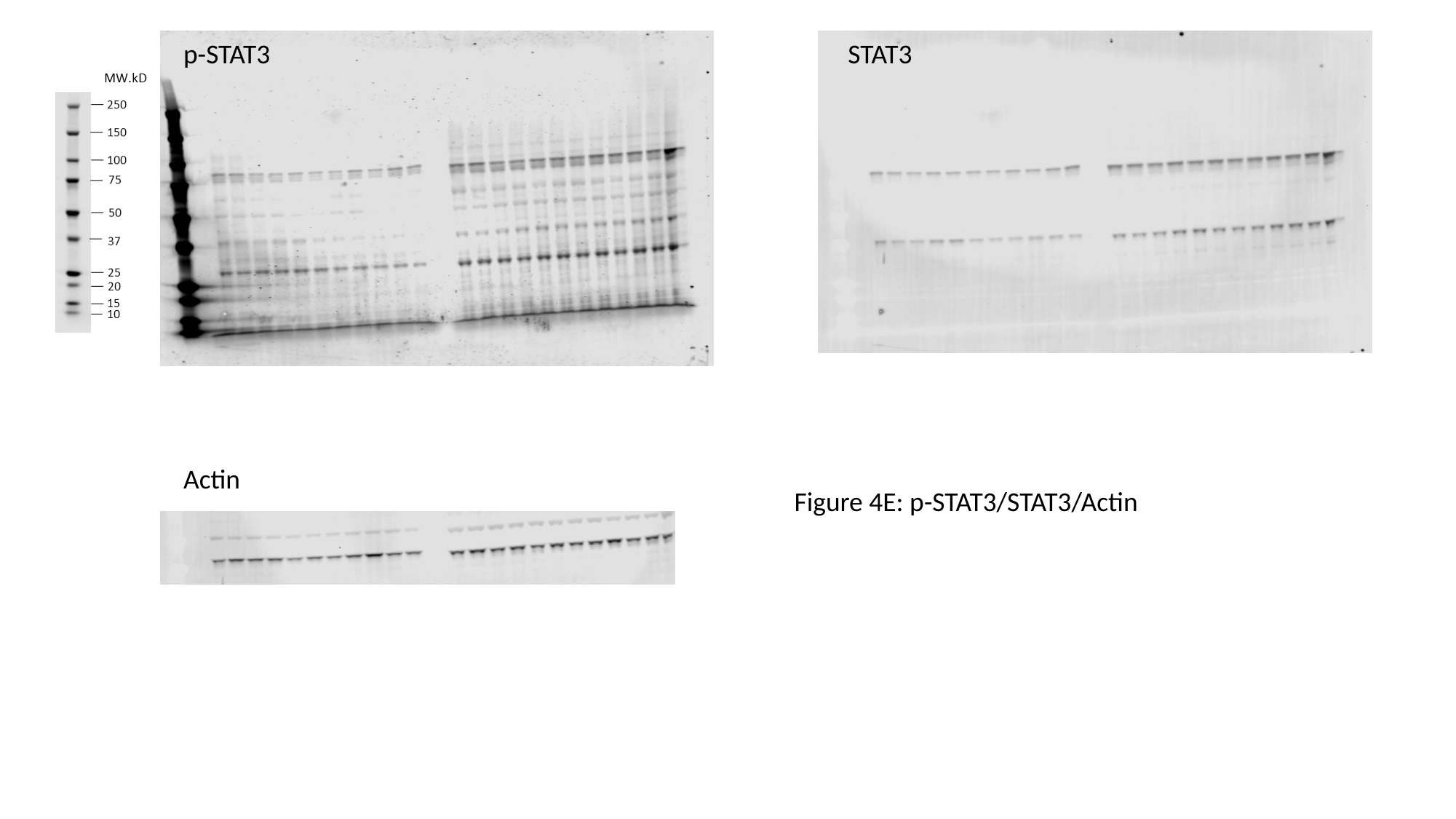

p-STAT3
STAT3
Actin
Figure 4E: p-STAT3/STAT3/Actin

## Slide 5
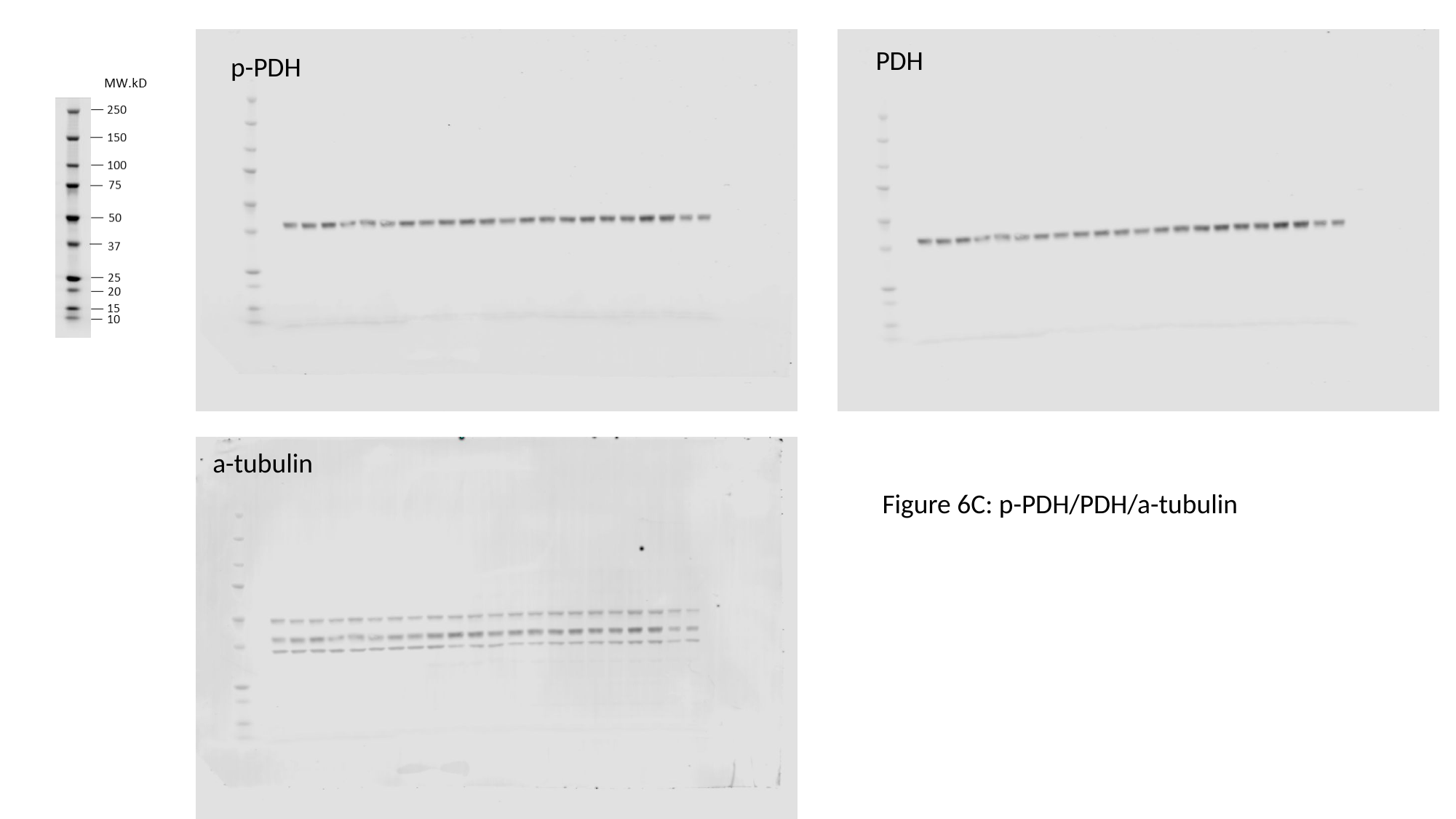

p-PDH
PDH
a-tubulin
Figure 6C: p-PDH/PDH/a-tubulin

## Slide 6
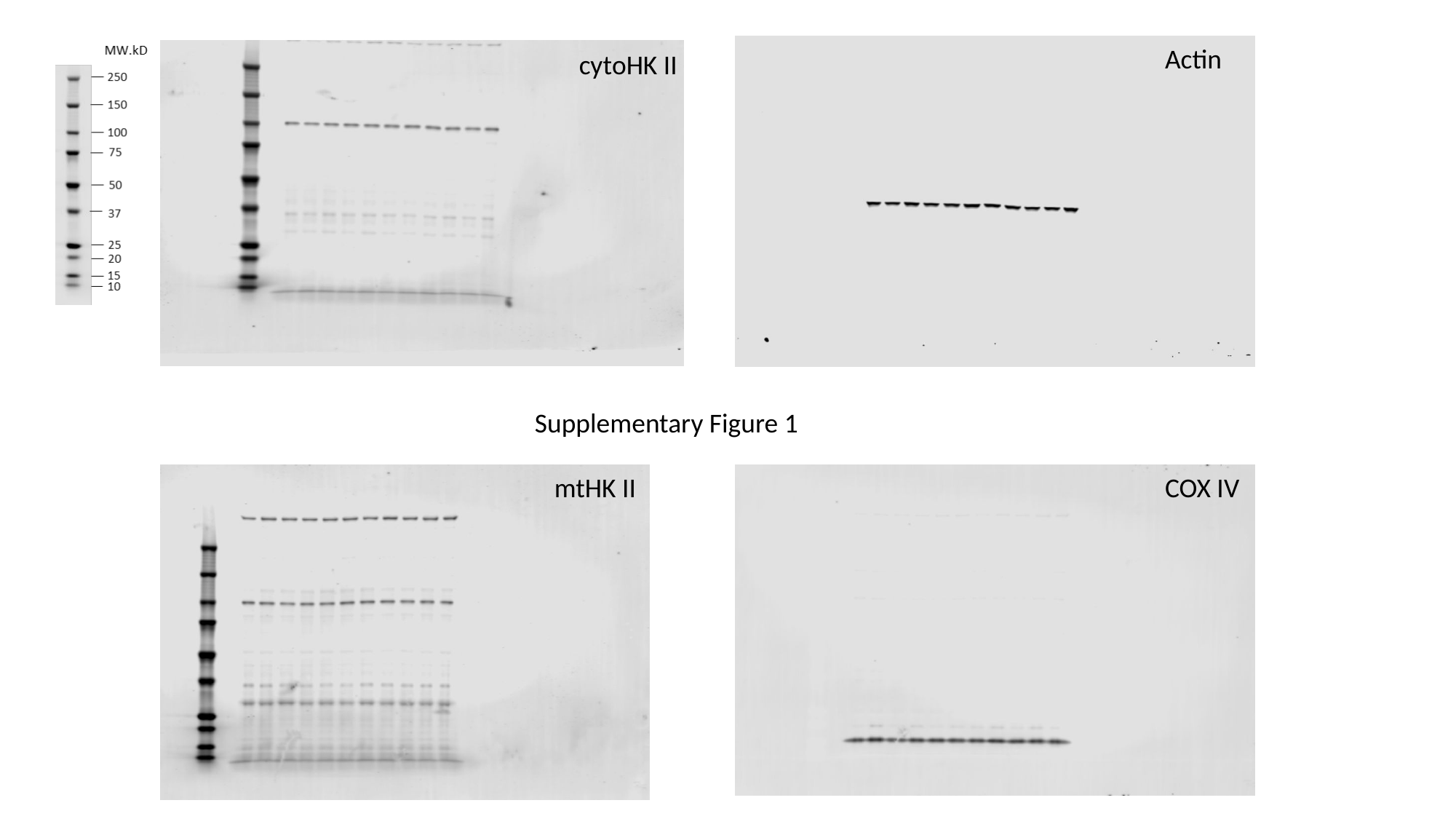

Actin
cytoHK II
Supplementary Figure 1
mtHK II
COX IV
