# Supplementary material for: NLRX1 Deletion Increases Ischemia-Reperfusion Damage and Activates Glucose Metabolism in Mouse Heart
Source: Front Immunol. 2020 Dec 11;11:591815. doi: 10.3389/fimmu.2020.591815 (PMC7759503; doi:10.3389/fimmu.2020.591815)
Supplement: Supplementary file 2 [file Table_1.docx]

Supplementary Material

## Supplementary Figures and Tables

**Supplementary Figure 1.** Survival signaling proteins in WT and NLRX1^-/-^ isolated hearts without Langendorff perfusion. **(A/B)** Representative immunoblots and analysis of total Akt and phospho-Akt; **(C/D)** Representative immunoblots and analysis of total AMPK and phospho-AMPK. Values represent mean ± SD (n=6/4 per group).


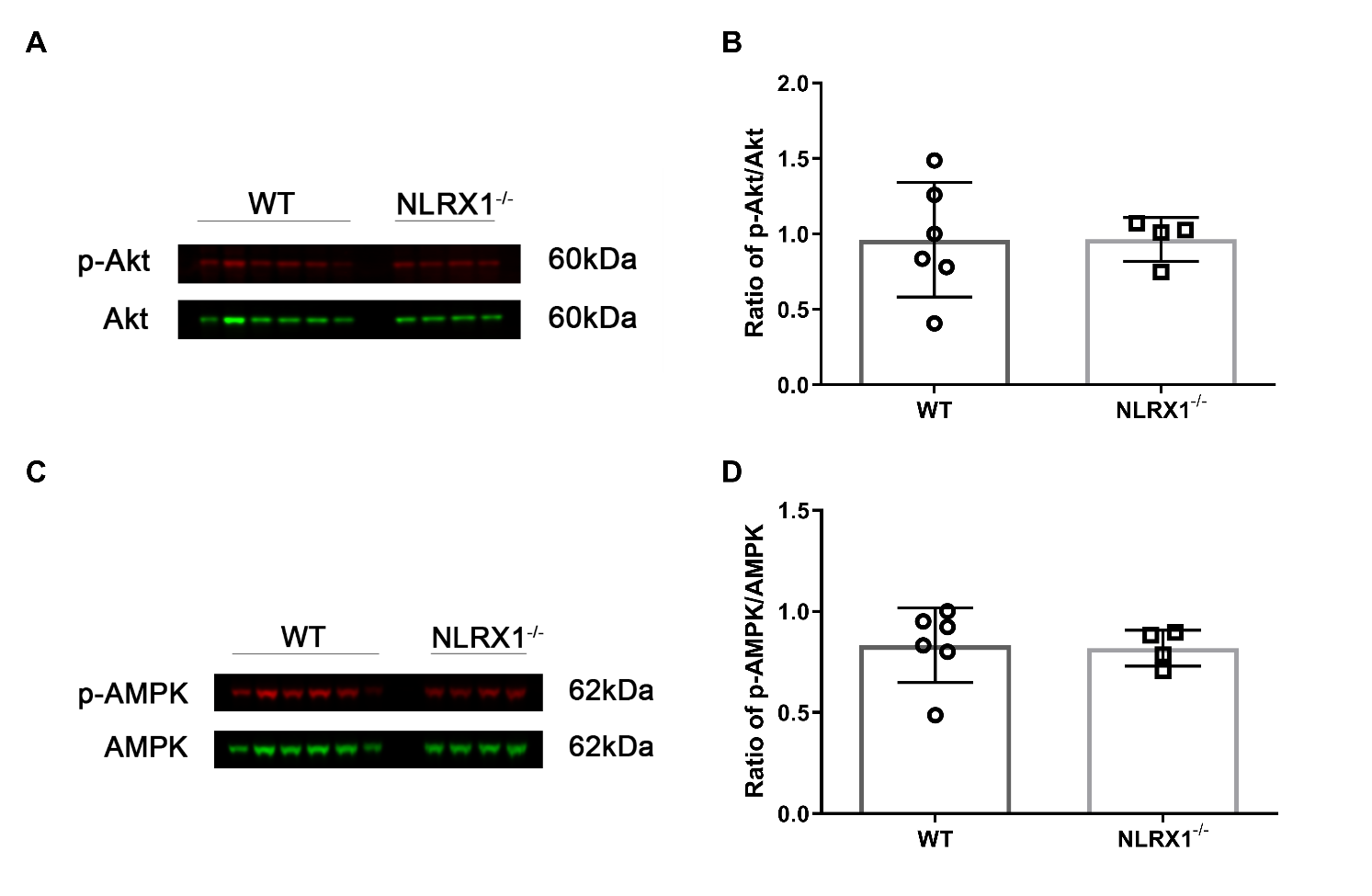


**Supplementary Figure 2.** Hexokinase (HK) activity, HK II expression and inflammatory factors at early stage of reperfusion in severe IR injury model of WT and NLRX1^-/-^ isolated mouse hearts of protocol 3. **(A/B)** HK activity in cytosolic fraction and mitochondrial fraction, respectively; **(C/E)** Representative immunoblots and analysis of HK II expression in cytosolic fraction; **(D/F)** Representative immunoblots and analysis of HK II expression in mitochondrial fraction; **(G/H)** IL-6 and TNFα expression by ELISA, respectively. CS, citrate synthase. Values represent mean ± SD. (n=6/5 per group).

**
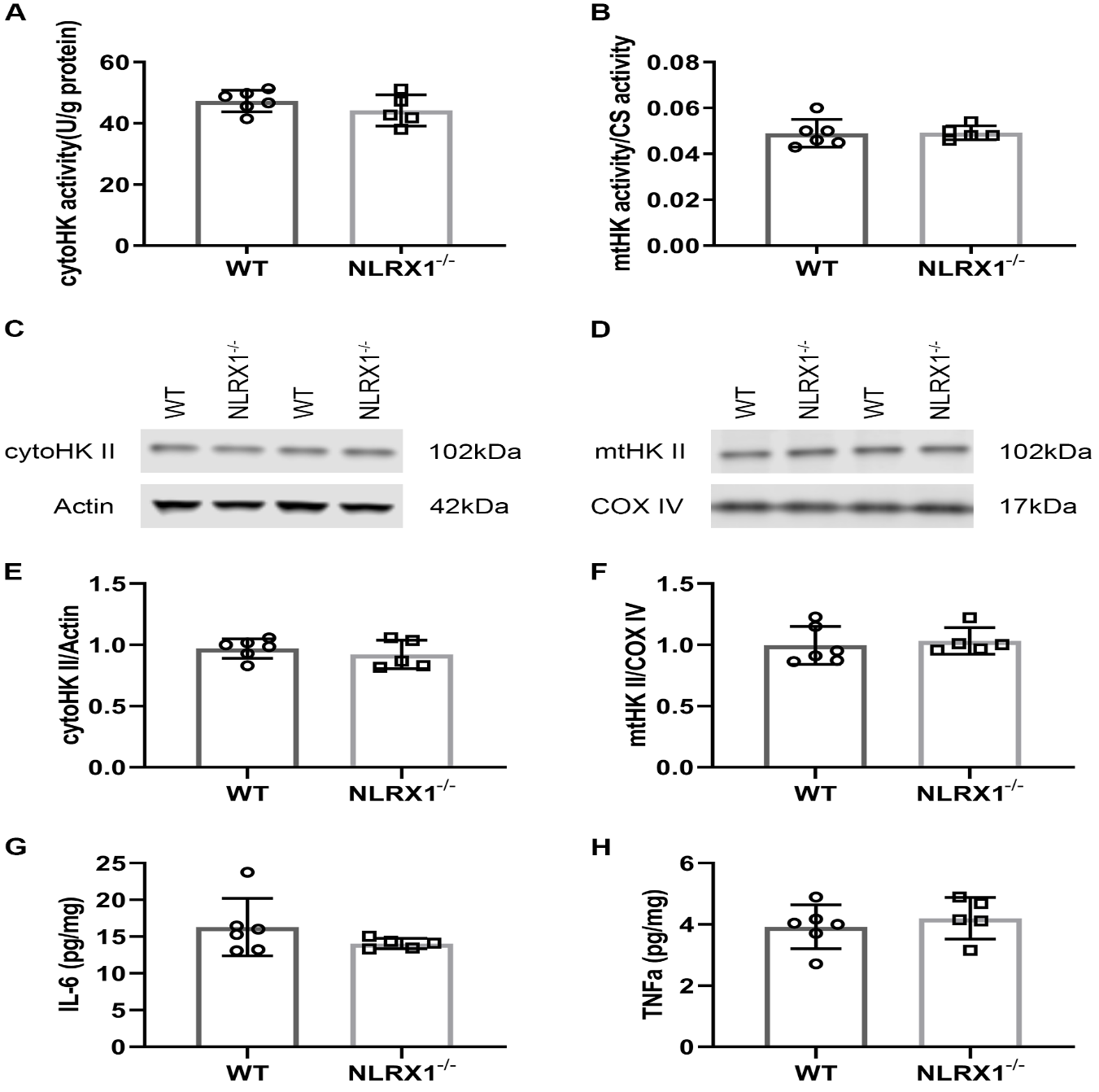
**

**Supplementary Table 1.** Derived equations to calculate the positional assignments of enrichments in metabolites from U-^13^C_6_ glucose metabolism of Langendorff perfused hearts. APE, atomic percent enrichment.

| **Metabolite** | **APE** |
| --- | --- |
| HexoseP_6 | [^13^C_6_] Hexose-6P |
| Erythrose4P_4 | [^13^C_4_] Erythrose-4P |
| PEP_3 | [U ^13^C_3_] PEP |
| Pyruvate_3 | [U ^13^C_3_] Pyr |
| [^13^C_2_]mtAcCoA (1) | 1-([^13^C_1_]Cit/[^13^C_1_]Mal) |
| [^13^C_2_]mtAcCoA (2) | ([^13^C_2_]Cit-[^13^C_2_]Mal)/ ([^13^C_0_]Mal-[^13^C_2_]Mal) |
| [^13^C_2_]mtAcCoA (4) | ([^13^C_4_]Cit-[^13^C_4_]Mal)/ ([^13^C_2_]Mal-[^13^C_4_]Mal) |
| [^13^C_2_]mtAcCoA (5) | [^13^C_5_]Cit/[^13^C_3_]Mal |
| [^13^C_2_]mtAcCoA (6) | [^13^C_6_]Cit/[^13^C_4_]Mal |
| [(4,5) ^13^C_2_]Citrate | [∑ ^13^C_2,3,4,5,6_]Citrate-[∑ ^13^C_2,3,4_]Mal*(1-[^13^C_2_]AcCoA) |
| [(4,5) ^13^C_2_] αKG | (φ_Cit🡪αKG_)**_*_**[(4,5) ^13^C_2_]Citrate |
| [(1,2)(3,4) ^13^C_2_]Mal | [^13^C_2_]Mal*([(4,5) ^13^C_2_]αKG/[^13^C_2,3,4,5_]αKG) |

**Supplementary Table 2.** Cohorts of positional enrichments in the flux contributions from metabolite precursor (denominator) to product (numerator) of U-^13^C_6_ glucose or U-^13^C_16_ palmitate perfused hearts.

| **φ** | **Numerator** | **Denominator** |
| --- | --- | --- |
| **φ_Hex-P🡪PEP_** | [U ^13^C_3_] PEP | [U ^13^C_6_] Hexose-6-phosphate |
| **φ_PEP🡪Pyr_** | [U ^13^C_3_] Pyr | [U ^13^C_3_] PEP |
| **φ_Pyr🡪AcCoA_** | [^13^C_2_] mtAcetylCoA | [U ^13^C_3_] Pyr |
| **φ_AcCoA🡪Cit_** | [∑ ^13^C_2,3,4,5,6_]Citrate-[∑ ^13^C_2,3,4_]Mal*(1-[^13^C_2_]AcCoA) | [^13^C_2_] mtAcetylCoA |
| **φ_Cit🡪αKG_** | [∑^13^C_1,2,3,4,5_] αKG | [∑ ^13^C_1,2,3,4,5,6_] Citrate |
| **φ_KG 🡪Fum_** | [∑ ^13^C_1,2,3,4_] Fum | [∑ ^13^C_1,2,3,4,5_] αKG |
| **φ_Fum🡪Mal_** | [∑ ^13^C_1,2_] Mal | [∑ ^13^C_1,2_] Fum |
| **φ_PEP🡪Lac_** | [U ^13^C_3_] Lac | [U ^13^C_3_] PEP |
| **φ_butCoA🡪AcCoA_** | [^13^C_2_] AcetylCoA | [∑ ^13^C_4_] butCoA |
| **φ_butCoA🡪Cit_** | [∑ ^13^C_2,3,4,5,6_]Citrate-[∑ ^13^C_2,3,4_]Mal*(1-[^13^C_2_]AcCoA) | [∑ ^13^C_4_] butCoA |
